# Evolutionary Dynamics of Plant TRM6/TRM61 Complexes

**DOI:** 10.3390/plants14121778

**Published:** 2025-06-11

**Authors:** Wenjie Yue, Tong Chen, Shuyi Liu, Xiaowen Shi

**Affiliations:** College of Agriculture and Biotechnology, Zhejiang University, Hangzhou 310058, China; 22416122@zju.edu.cn (T.C.); 22416202@zju.edu.cn (S.L.); xiaowenshi@zju.edu.cn (X.S.)

**Keywords:** m^1^A methylation, TRM6 and TRM61 homologs, evolution, wheat, expression profiles

## Abstract

N^1^-methyladenosine (m^1^A) serves as a critical regulatory modification in plant mRNA. In *Arabidopsis*, the TRM61/TRM6 complex functions as m^1^A58 methyltransferase writers essential for organogenesis, reproduction, and hormonal signaling. However, the evolutionary dynamics of the TRM61/TRM6 complex across plant lineages remain poorly understood. In this study, we systematically identified TRM6 and TRM61 homologs across 306 plant species and uncovered the conserved evolutionary trajectories between them. These two methyltransferase subunits retain conserved structural motifs, respectively, and exhibit coordinated expression patterns in plants. In wheat (*Triticum aestivum* L.) and its progenitors, TRM6 and TRM61 proteins demonstrate polyploidization-associated evolutionary coordination. Their promoters harbor stress-, light-, and hormone-responsive *cis*-elements. Furthermore, the *TRM6* and *TRM61* genes in wheat exhibit diverse expression profiles across developmental tissues and under abiotic stress conditions. The differences in allelic frequency among *TRM6* and *TRM61* variants between wild and domesticated wheat populations suggest that they may have undergone selection during wheat domestication and improvement. This study provides an evolutionary framework for the TRM61/TRM6 complex.

## 1. Introduction

Originally proposed by Francis Crick in 1958, the Central Dogma of molecular biology describes the unidirectional flow of genetic information from DNA through RNA to proteins. The discovery of reverse transcription, the RNA-templated synthesis of DNA, expands this framework, thereby solidifying its role as the cornerstone of molecular genetics [1]. As pivotal mediators of epigenetic regulation, RNA modifications orchestrate gene expression without altering DNA sequences, forming a pervasive regulatory network in genetic information flow that adds complexity to transcriptional and post-transcriptional processes [2]. Breakthroughs in RNA modification detection, spanning liquid chromatography followed by mass spectrometry (LC-MS) and high-throughput sequencing, have facilitated the functional annotation of over 170 distinct RNA chemical modifications, while also elucidating their spatiotemporal regulation through conserved enzymatic cascades [3,4,5]. These modifications can be broadly classified into four major chemical categories: (1) direct covalent modifications of nucleobases (e.g., N^6^-methyladenosine (m^6^A)), (2) noncanonical base rearrangements (e.g., pseudouridylation), (3) 2′-O-methylation of the ribose (Nm), and (4) other more complex hybrid modifications (e.g., 5-hydroxymethylcytosine) [2]. Together, these modifications precisely modulate RNA metabolism by exerting control over the molecular stability, subcellular transport processes, and translational accuracy [6].

RNA modifications are dynamically regulated by the evolutionarily conserved “Writer-Reader-Eraser” triad. In this system, writers (e.g., METTL3 for m^6^A), erasers (e.g., FTO for m^6^A), and readers (e.g., YTHDF2 for m^6^A) collaboratively establish, interpret, and remove chemical marks, respectively [7,8]. Emerging evidence indicates that covalent RNA modifications modulate RNA behavior by altering their structural conformations, translational activity, functional interactions, and degradation pathways [2,9]. These modifications are widely distributed across plant tRNAs, rRNAs, and mRNAs, which regulate RNA processing and function through evolutionarily conserved mechanisms [2]. Among them, m^6^A is the first and most abundant internal modification discovered on mRNA, and it has been found in viruses and eukaryotes, such as yeasts, mammals, insects, and plants [10,11,12]. m^6^A is predominantly enriched around the stop codon and in the 3′ untranslated region (UTR) of mRNA transcripts and can affect multiple aspects of RNA metabolism (e.g., RNA splicing, localization, translation, and stability), thereby modulating the gene expression in many biological processes [13,14]. For instance, cytoplasmic m^6^A methylation has been shown to stabilize photosynthesis in plants under cold environmental conditions [15]. In animals, m^6^A also plays a critical role in early development and embryogenesis [16,17].

N^1^-methyladenosine (m^1^A) was first discovered in 1961 and has since been found in tRNAs, rRNAs, and mRNAs [18,19,20,21]. Similar to m^6^A, m^1^A is an ancient and evolutionarily conserved modification through archaea, bacteria, and eukarya, which host methylation at the N^1^ position of adenosine that occurs on the Watson–Crick interface and generates a positively charged base [21,22,23]. In mRNAs, m^1^A is enriched around the start codon upstream of the first splice site and may affect translation by promoting non-canonical binding of the exon-exon junction complex at 5′ UTRs devoid of 5′ proximal introns [23,24]. m^1^A is now recognized as a widespread mRNA modification in plants, with its roles in RNA metabolism and plant growth and development being actively investigated [25,26,27,28]. The advent of m^1^A sequencing technology, such as m^1^A-ID-seq, has propelled the comprehensive exploration of m^1^A in plants [21]. In the flowering plant, petunia (*Petunia hybrida*), a transcriptome-wide study identified 4993 m^1^A peaks in 3231 genes expressed in corollas [25]. In dinoflagellates, a group of unicellular eukaryotic phytoplankton, m^1^A, rather than m^6^A, represents the most abundant internal mRNA modification, likely regulating the expression of metabolism-related genes via translational control [29]. The biological functions of m^1^A in plants are beginning to be elucidated. For example, m^1^A sites exhibit dynamic methylation changes in response to ethylene treatment and during leaf development in petunia [25]. In tomato (*Solanum lycopersicum* L.), m^1^A modifications are not only widespread in mRNAs, but also detected in long non-coding RNAs and circular RNAs, potentially influencing the expression of the genes involved in the ripening process [28].

The identification of the RNA-binding proteins associated with specific mRNA modifications is critical for elucidating their biological roles and context-dependent regulatory mechanisms. With the accumulating data on RNA modifications and their associated “Writer-Reader-Eraser” systems, the molecular mechanisms and biological functions of m^6^A methylation in plants have been comprehensively characterized [2,30,31]. In contrast, m^1^A modifications in plants remain largely unexplored. In mammals, YTH domain-containing proteins serve as readers for both m^6^A and m^1^A, while ALKBH1 and ALKBH3 function as m^1^A erasers on mRNAs [32,33]. However, plant-specific m^1^A readers and erasers remain unidentified. In eukaryotes, the writer of m^1^A modification is catalyzed by the TRM6/TRM61 holoenzyme [34,35]. In *Saccharomyces cerevisiae*, two TRM6/TRM61 heterodimers assemble into a heterotetramer structure [36]. In *Magnaporthe oryzae*, this complex mediates dynamic m^1^A methylation at position 58 of tRNAs to modulate the translation during both initiation and elongation [37]. The evolutionary conservation of RNA modification machinery across biological kingdoms has provided a framework for dissecting the mechanisms of m^1^A methylation in plants. In *Arabidopsis*, the homologs of yeast TRM61 and TRM6 protein (nucleus-localized complex AtTRM61/AtTRM6) have been characterized as the tRNA m^1^A58 methyltransferase heterodimer, and deficiency in either *AtTRM61* or *AtTRM6* leads to embryo arrest and seed abortion due to disrupted tRNAi^Met^ stability [26,27]. Preliminary evidence suggests that m^1^A methylation plays a critical role in modulating plant organogenesis, reproductive processes, and hormone signaling pathways through dynamic RNA stability and translation efficiency control [25,26,27]. However, the molecular mechanisms underlying m^1^A methylation and its functional roles in plant development and physiology remain largely unknown, representing critical knowledge gaps in plant epitranscriptomics. Evolutionary analyses of RNA modification writers, readers, and erasers in plants provide critical insights into the potential functional diversification and mechanisms of RNA modifications [38]. Investigating the evolutionary trajectory of the *TRM6* and *TRM61* genes in plants is essential for understanding the functional architecture of m^1^A methylation and its regulatory roles in organogenesis and reproductive success. The advent of high-throughput sequencing technologies has driven a rapid expansion of plant genomic datasets, enabling systematic phylogenetic investigations into the evolutionary trajectory of m^1^A writer (TRM61/TRM6 complex) in plants from a macroevolutionary perspective. Bread wheat (2n = 6x = 42, *Triticum aestivum* L. (AABBDD genome)), an allohexaploid that arose through two hybridization events involving three diploid ancestors, serves as an ideal model for investigating polyploid evolution and speciation [39,40,41].

To explore the macroevolutionary dynamics of the TRM61/TRM6 complexes in plants, we identified the orthologs of TRM6 and TRM61 across 306 plant species, encompassing major lineages including algae, Streptophyta, Bryophyta, and Tracheophyta. We examined lineage-specific expansion patterns and potential functional diversification of these two m^1^A methyltransferase subunits. In addition, we investigated the evolutionary dynamics of the TRM6 and TRM61 homologs in the wheat lineage to assess their functional contributions to m^1^A modification and to understand the subgenome-specific adaptive divergence among the A, B, and D subgenomes during domestication and breeding. Our study provides valuable insights into m^1^A modification in plants by reconstructing the evolutionary trajectory and functional divergence of m^1^A methyltransferases (TRM61/TRM6), which may help in the elucidation of the molecular mechanisms underlying m^1^A modification in plants.

## 2. Results

### 2.1. Identification of TRM6 and TRM61 Families in Plants

**Figure 1:**
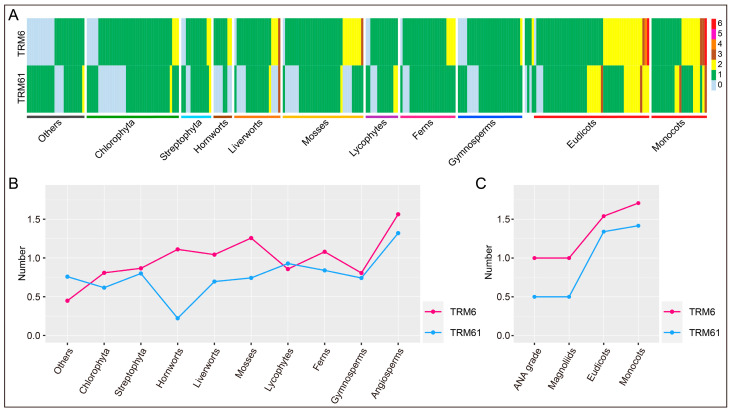
The distribution of TRM6 and TRM61 homologs in plants. (**A**) The number of *TRM6s* and *TRM61s* identified in each species. The color represents the gene number. The category “Others” encompasses algal taxa not classified under Chlorophyta (green algae), including phylogenetically distinct lineages such as Cryptophyceae, Glaucophyta, Ochrophyta, and Rhodophyta. (**B**) The average number of genes in the *TRM6* and *TRM61* families within each plant lineage. (**C**) The average number of genes in the *TRM6* and *TRM61* families in angiosperms. The y-axis denotes the mean gene number per lineage, calculated by dividing the total number of genes identified in a lineage by the number of species that it contains.

To investigate the distribution of *TRM6s* and *TRM61s* across the plant kingdom, we collected the proteome of 306 species spanning major lineages, including angiosperms, gymnosperms, ferns, lycophytes, mosses, liverworts, hornworts, and algae for gene identification (Figure S1 and Supplementary Data S1). A total of 328 *TRM6* and 267 *TRM61* homologs were identified from 285 species. Notably, 21 species with neither *TRM6* nor *TRM61* were identified, and most of these species were algae, with a few sporadically distributed among gymnosperms, ferns, liverworts, and hornworts (Supplementary Data S2). Interestingly, *TRM6s* and *TRM61s* are not universally co-retained across species. Among the analyzed taxa, 86 species possess only one type of homolog (with the number of *TRM6*:*TRM61* = 0:n or n:0), indicating lineage-specific gene loss events or the evolution of compensatory m^1^A regulatory mechanisms (Figure 1A and Supplementary Data S2). To assess the gene family expansion of *TRM6s* and *TRM61s* during plant diversification, we compared their copy numbers across different plant lineages. On average, the number of *TRM6s* increased from algae to Bryophyta (mosses, liverworts, and hornworts), decreased in lycophytes and gymnosperms, and then expanded in angiosperms. In contrast, *TRM61s* were reduced in hornworts but also expanded in angiosperms (Figure 1B). Furthermore, we observed that *TRM6* and *TRM61* expansion was more pronounced in monocots than in eudicots (Figure 1C). Overall, the *TRM6* gene family exhibited a greater degree of expansion than *TRM61* across plant lineages (Figure 1). These findings reveal that TRM6 and TRM61 homologs are widely distributed in algae, bryophytes, and vascular plants, with remarkable expansion occurring in angiosperms. Notably, these two gene families display divergent distribution patterns among these major plant groups.

### 2.2. The Evolution of TRM6 and TRM61 Families in Plants

**Figure 2:**
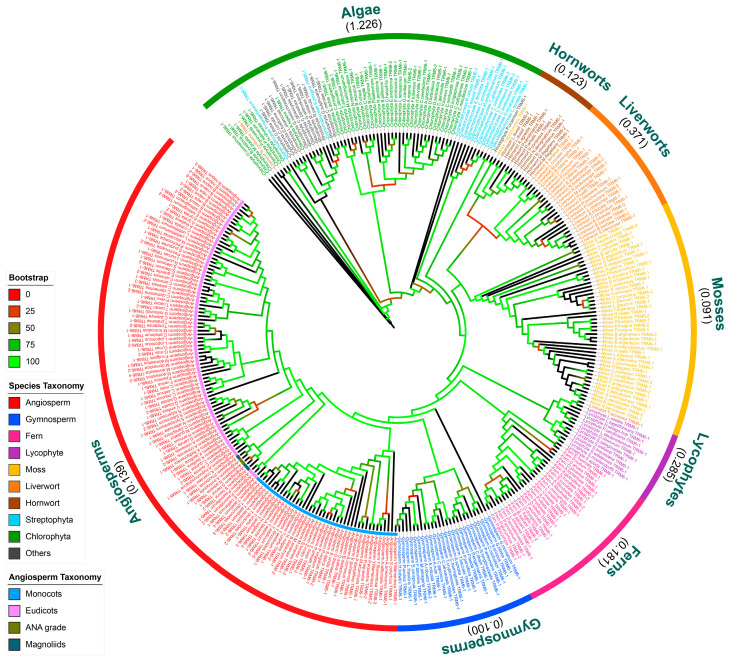
The evolution of TRM6 homologs in plants. The identification of TRM6 homologs in plants is detailed in the Section 4. All identified TRM6 protein sequences were aligned using PRANK and subsequently trimmed with Trimal (−gt 0.1). A maximum likelihood phylogenetic tree was constructed using IQ-TREE 2 with the best-fit model Q.plant + R7, with 1500 bootstrap replicates. The color of the branches corresponds to the support bootstrap values. The average branch length for each taxon, calculated after excluding the extremely short branches (defined as having a length < 0.001), is shown in parentheses below the taxon name. Four subclasses of angiosperms are indicated by the stripe colors at the top of the branches. Taxonomy classifications are listed in Supplementary Data S1, with different font colors used to distinguish the taxa. The category “Others” encompasses algal taxa not classified within Chlorophyta (green algae), including phylogenetically distinct lineages such as Cryptophyceae, Glaucophyta, Ochrophyta, and Rhodophyta. All identified TRM6 homologs are listed in Supplementary Data S2.

**Figure 3:**
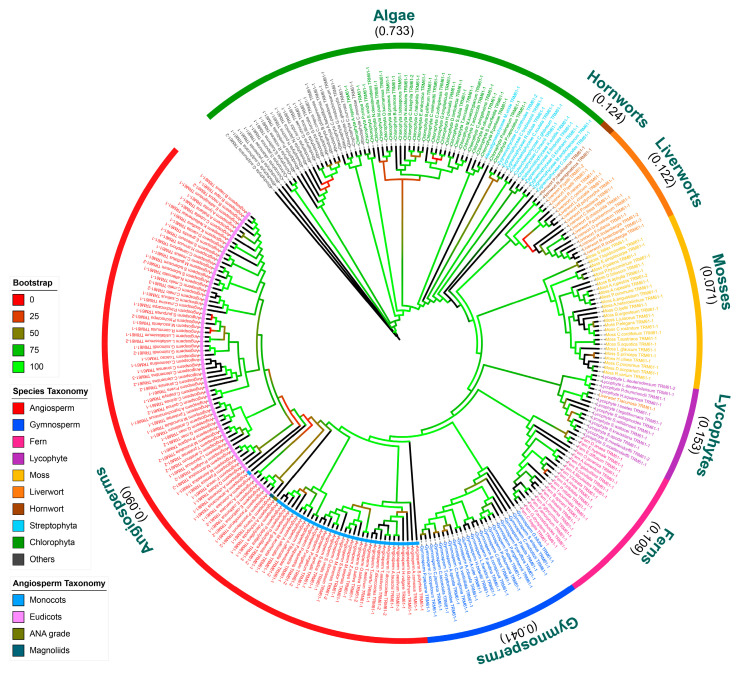
The evolution of TRM61 homologs in plants. The phylogenetic tree of TRM61 proteins was constructed following the same approach as described in Figure 2.

To investigate the evolutionary trajectory of *TRM6s* and *TRM61s* in plants, we conducted a comprehensive phylogenetic analysis of all the identified homologs. Overall, the results demonstrate that the *TRM6* and *TRM61* families exhibit a similar evolutionary trajectory across plant lineages. These two families evolved from algal lineages to Bryophyta (hornworts, liverworts, mosses), diversified further in lycophytes, ferns, and gymnosperms, and ultimately underwent lineage-specific expansion in angiosperms (Figure 2 and Figure 3, S2 and S3). To assess the degree of sequence divergence within these families, we calculated the average genetic distances of TRM6 and TRM61 proteins within each plant taxon, respectively (Figure 2 and Figure 3, S2 and S3). Although the sequence divergence varied among taxa, the average genetic distances of the TRM6 and TRM61 proteins exhibited concordant variation patterns across the plant taxa (Pearson correlation coefficient (PCC) = 0.989 and *p* value < 0.01). These results suggest that while the degrees of variation in TRM6 and TRM61 proteins differ across the plant taxa, the two gene families have followed parallel evolutionary trajectories throughout plant diversification. Notably, the divergence levels of TRM6 sequences are higher than those of TRM61 homologs in liverwort and lycophyte taxa, suggesting relatively weak correlations between the local variation patterns of these two gene families.

The rapid diversification of angiosperms was accompanied by the expansion of the *TRM6* and *TRM61* gene families. Our phylogenetic analysis revealed a clear divergence between the eudicots and monocots in both families (Figure 2 and Figure 3). These findings indicate the functional conservation and coordinated evolutionary trajectories of the *TRM6* and *TRM61* families during angiosperm radiation, potentially driven by conserved selective pressures or co-evolutionary constraints.

### 2.3. Conserved TRM6 and TRM61 Homologs Show Similar Expression Profiles in Plants

**Figure 4:**
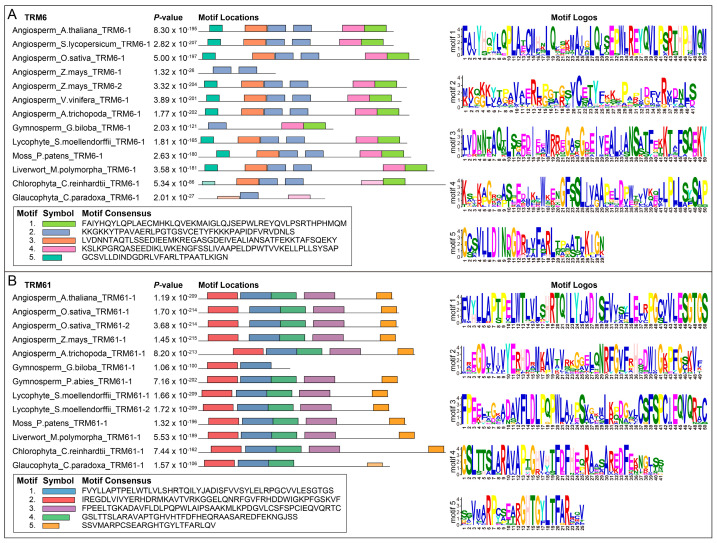
Comparison of conserved domains in (**A**) TRM6 and (**B**) TRM61 homologs from 13 phylogenetically representative species. The gene nomenclature method is described in the Section 4. The numeric range of the suffix for each gene name reflects the number of *TRM6* or *TRM61* genes identified in the species. Protein sequences of TRM6 and TRM61 homologs were submitted to the MEME suite for motif identification. The *p*-value denotes the probability that a random sequence (with the same length and conforming to the background) would have position *p*-values such that the product is less than or equal to the value calculated for the sequence under test. Abbreviation of species names: *Arabidopsis thaliana* (*A. thaliana*); *Solanum lycopersicum* (*S. lycopersicum*); *Oryza sativa* (*O. sativa*); *Zea mays* (*Z. mays*); *Vitis vinifera* (*V. vinifera*); *Amborella trichopoda* (*A. trichopoda*); *Ginkgo biloba* (*G. biloba*); *Picea abies* (*P. abies*); *Selaginella moellendorffii* (*S. moellendorffii*); *Physcomitrium patens* (*P. patens*); *Marchantia polymorpha* (*M. polymorpha*); *Chlamydomonas reinhardtii* (*C. reinhardtii*); *Cyanophora paradoxa* (*C. paradoxa*).

To investigate the evolutionary dynamics of conserved motifs in the TRM6 and TRM61 proteins across plants, we extracted the protein sequences of both families from 11 phylogenetically representative land plants and two algae for conserved motif discovery. Our findings demonstrated that the protein sequences of TRM6s and TRM61s are conserved across most land plants, as evidenced by the consistent number and types of conserved motifs identified (Figure 4). Notably, *Ginkgo biloba* exhibits relatively fewer conserved motifs in both TRM6 and TRM61 proteins compared to other land plants, suggesting substantial functional divergence during long-term plant evolution. Furthermore, compared to *Cyanophora paradoxa*, the TRM6 and TRM61 proteins in *Chlamydomonas reinhardtii* exhibit greater motif similarity to their orthologs in land plants, suggesting stronger evolutionary constraints on these methyltransferase subunits in the green lineage (Figure 4).

**Figure 5:**
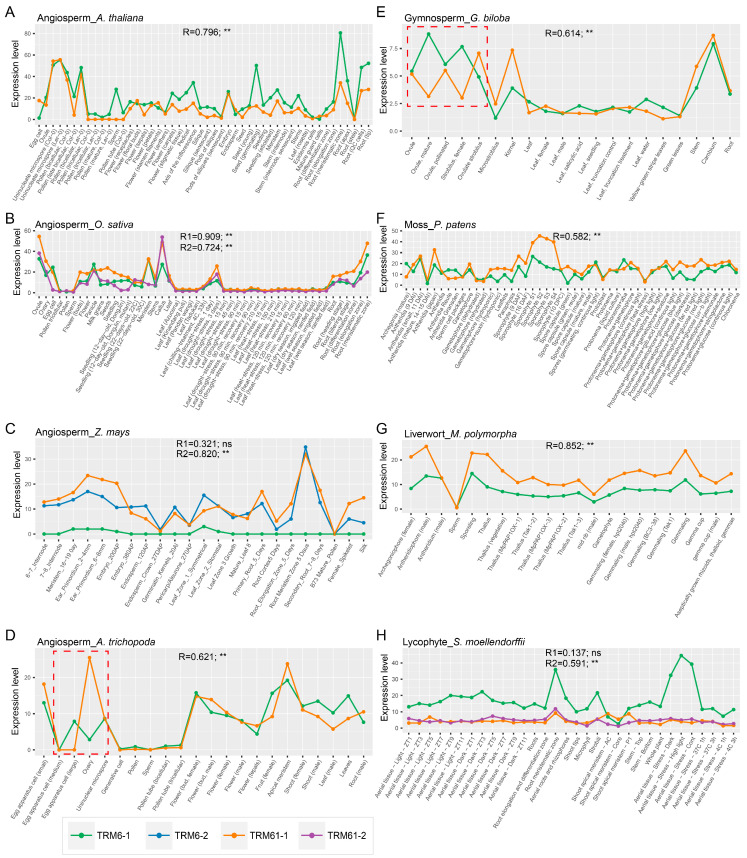
The expression level correlation of *TRM6s* and *TRM61s* during plant organogenesis and reproduction processes (**A**–**H**). The expression levels (in Transcripts Per Million (TPM)) of *TRM6s* and *TRM61s* for various organs and gametes of plant species, including algae, bryophytes, vascular plants, gymnosperms, and flowering plants, were obtained from the EVOREPRO (www.evorepro.plant.tools (accessed on 27 February 2025)) and MaizeGDB (https://maizegdb.org/ (accessed on 27 February 2025)) databases. For each species, Pearson correlation was performed to assess the relationship between *TRM6* and *TRM61* expression levels under diverse conditions. R stands for the PCC. The *p* value for Pearson correlation is defined as follows: ** *p* value < 0.01. R1 and R2 denote the PCC for *TRM6-1* vs. *TRM61-1* and *TRM6-1* vs. *TRM61-2* (or *TRM6-2* vs. *TRM61-1*) pairs, respectively. Numeric suffixes (e.g., −1, −2) correspond to gene copy numbers within each gene family. The local opposite expression trends in *Amborella trichopoda* and *Ginkgo biloba* are highlighted with red dashed boxes. The expression level of *TRM6* and *TRM61* genes across different species is presented in Supplementary Data S3.

The TRM61/TRM6 methyltransferase complex is essential for embryogenesis and endosperm formation of *Arabidopsis*, as demonstrated by the embryo arrest and seed abortion phenotypes observed in both *AtTRM61* and *AtTRM6* loss-of-function mutants [26,27]. To investigate the role of this m^1^A writer in organogenesis and reproduction across land plants, we analyzed the expression patterns of both gene families in the aforementioned 13 species and evaluated the expression level correlation between *TRM6* and *TRM61* using Pearson correlation analysis. Our results demonstrated that the expression levels of *TRM6s* and *TRM61s* are significantly and positively correlated (PCC > 0.5 and *p* value < 0.05) across diverse organs in most of the land plants examined (Figure 5). Furthermore, spatiotemporal expression profiling across land plants demonstrated that *TRM6* and *TRM61* orthologs exhibit higher expression levels in reproductive organs (male and female organs) and root meristems (Figure 5 and S4, and Supplementary Data S3), suggesting that these two proteins may cooperate to function in the organogenesis and reproduction processes of land plants. In addition, we also detected local opposite expression trends between TRM6 and TRM61 homologs in the female reproductive organs of *Amborella trichopoda* (a basal angiosperm) and *Ginkgo biloba* (a gymnosperm) (Figure 5D,E), implying potential lineage-specific regulatory divergence of the TRM61/TRM6 complex during plant evolution. This expression of antagonism may reflect a dynamic rewiring of the m^1^A methylation machinery, potentially driving functional innovations in angiosperm female gametophyte development and marking a key transition in reproductive strategy between gymnosperms and angiosperms. Furthermore, we observed significant positive correlations (*p* value < 0.05) in expression levels between *TRM6s* and *TRM61s* under diurnal rhythm changes, nutrient deficiencies, and various stress conditions in both *Cyanophora paradoxa* and *Chlamydomonas reinhardtii* (Figure S4A,B). Collectively, these results suggest that the TRM6 and TRM61 homologs have coevolved and may play coordinated roles in plant organogenesis, reproduction, and environmental adaptation.

### 2.4. The Evolutionary Dynamics of TRM6 and TRM61 Homologs in the Wheat Lineage

**Figure 6:**
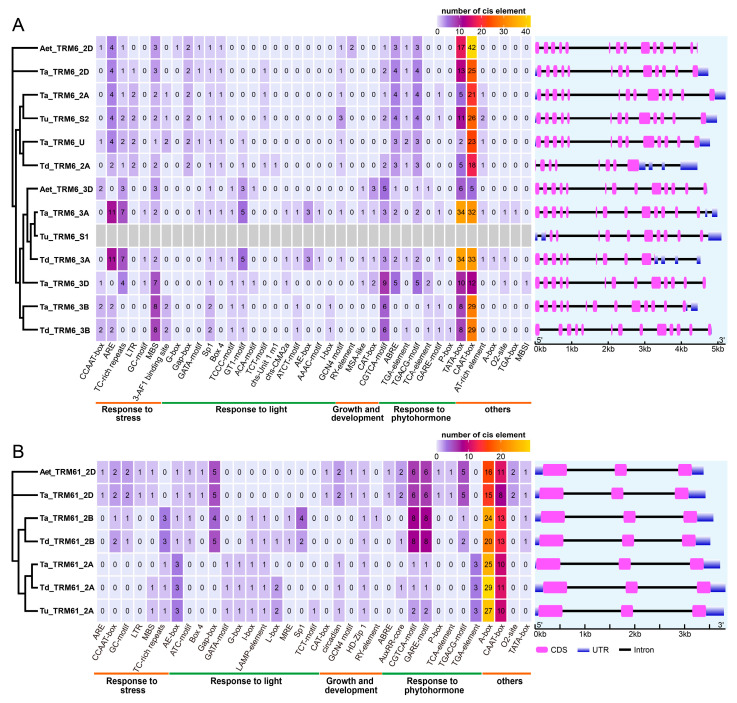
The promoter *cis*-acting regulatory elements (CREs) and gene structure of (**A**) *TRM6s* and (**B**) *TRM61s* in wheat and its diploid and tetraploid progenitors. CREs were analyzed within the 2000 bp promoter regions of TRM6 and TRM61 homologs from wheat, *T. dicoccoides*, *T. urartu*, and *A. tauschii* using the PlantCARE database (accessed on 3 March 2025). The color scale indicates the number of CREs. Since the promoter sequence of *Tu_TRM6_S1* is unavailable, the CREs cannot be analyzed and are marked in gray. The gene structures are shown in the right panel.

The well-characterized evolutionary trajectory of allohexaploid wheat provides a highly tractable genomic system for investigating the molecular evolutionary mechanisms underlying de novo speciation events in angiosperms. To explore the evolution of the TRM61/TRM6 complex during this process, we extracted the identified *TRM6s* and *TRM61s* in wheat and its diploid (2n  =  2x  =  14, *Triticum urartu* (AA genome) and *Aegilops tauschii* (DD genome)) and tetraploid (2n  =  4x  =  28, *Triticum dicoccoides* (AABB genome)) ancestral lineages. In total, 13 TRM6 homologs (*Ta*/*Td*/*Tu*/*Aet* = 6:3:2:2, compared to the expected 6:4:2:2 ratio) and 7 TRM61 homologs (3:2:1:1) were identified across the four species, revealing lineage-specific contraction dynamics of the m^1^A methyltransferase subunits during wheat polyploidization (Table 1). TRM6 homologs exhibited divergent biochemical properties and an exon–intron structure, suggesting that functional innovation may have occurred during wheat polyploidization (Figure 6A and Table 1). In contrast, TRM61 homologs showed strict conservation in both biochemical characteristics and gene structures across all four species, reflecting strong functional constraints associated with m^1^A catalytic activity (Figure 6B and Table 1). These results suggest that the m^1^A methyltransferase subunits possess evolutionary plasticity in the wheat lineage while maintaining their core functionality.

The phylogenetic analysis of TRM6 and TRM61 proteins across wheat and its progenitors revealed coordinated evolutionary trajectories aligned with wheat polyploidization events (Figure 6). To further explore the evolution of expression modulation of TRM6 and TRM61 homologs in Triticeae, we analyzed *cis*-acting regulatory elements (CREs) within the 2-kb promoter regions of TRM6 and TRM61 homologs across allohexaploid wheat and its diploid and tetraploid progenitors. Our results revealed widespread conservation of stress-responsive (e.g., LTR), light-regulated (e.g., Sp1), phytohormone-responsive (e.g., MeJA-related CGTCA-motifs), and developmental-related CREs in the promoter of these methyltransferase subunit genes, suggesting pleiotropic roles for m^1^A modification in plant adaptation (Figure 6). Notably, we observed subgenome-specific CRE retention patterns. For example, in the B and D subgenomes, the *TRM61* promoters retained eight and six MeJA-responsive CGTCA CREs, respectively, whereas in the A subgenome, only a single CGTCA motif was present in both wheat and *T. dicoccoides*, and two copies were found in *T. urartu* (Figure 6B). This asymmetric distribution of regulatory elements reflects the dynamic selection pressures acting during wheat allopolyploid speciation events, implying both functional conservation and regulatory plasticity in tRNA modification systems.

### 2.5. TRM6 and TRM61 Homologs Undergo Divergent Selection Pressures During Wheat Evolution and Breeding

**Figure 7:**
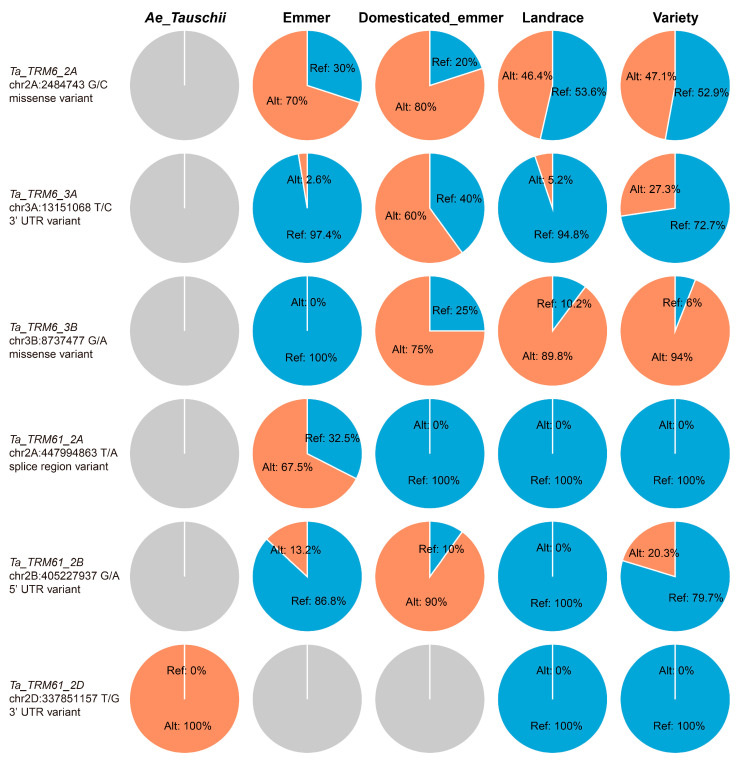
Selective footprint examples of *Ta_TRM6s* and *Ta_TRM61s* during wheat domestication and improvement. Single nucleotide polymorphism (SNP) variants (5′/3′ UTR, missense, splice regions) and their allele frequencies across five wheat ploidy groups were obtained from the Wheat Genome Variation Database (WGVD) (accessed on 11 March 2025). Each row of the pie charts illustrates the allele frequency distribution patterns of specific genetic variants across diverse wheat ploidy populations (diploid, tetraploid, and hexaploid). The SNP variant information of all *Ta_TRM6s* and *Ta_TRM61s* is presented in Supplementary Data S4. Ref: reference base; Alt: alternate base.

After being domesticated from diploid and tetraploid progenitors, hexaploid wheat was improved through modern breeding innovations for yield enhancement and climate resilience. To investigate whether the m^1^A methyltransferase subunits were subjected to selection during the domestication and improvement process of wheat, we systematically analyzed SNP variants within the 5′ UTRs, 3′ UTRs, missense coding regions, and splice regions of TRM6 and TRM61 homologs across diverse ploidy wheat populations (diploid, tetraploid, and hexaploid), integrating allele frequency spectra to identify selective footprints during wheat domestication and breeding (Supplementary Data S4). Among the *Ta_TRM6* genes, *Ta_TRM6_2D* and *Ta_TRM6_3D* showed minimal selection signals, with stable allele frequencies across the wheat evolutionary stages (Supplementary Data S4). In contrast, the remaining *Ta_TRMs* exhibited various selection patterns. For example, a missense variant (G/C allele, chr2A:2484743) in *Ta_TRM6_2A* displayed increasing allele frequency during tetraploid wheat breeding (wild emmer to domesticated emmer), suggesting positive selection. However, its frequency was subsequently decreased in the hexaploid wheat populations (landrace and variety), indicating purifying selection during the domestication process of wheat. In addition, a 3′ UTR variant (T/G allele, chr2D: 337851157) in *Ta_TRM61_2D* was depleted during the domestication process of hexaploid wheat (Figure 7). The allelic frequency dynamics of *TRM6* and *TRM61* genes during wheat domestication and improvement appear to correlate with the potential involvement of m^1^A methylation in mediating environmental adaptation processes and yield-related trait optimization.

To further explore the divergent evolutionary trajectories of *TRM6s* and *TRM61s*, we performed a comprehensive micro-collinearity analysis of TRM6 and TRM61 homologs among Triticeae species (wheat and *Hordeum vulgare*), other Poaceae species (*Oryza sativa*, *Zea mays*, and *Sorghum bicolor*), and a representative eudicot (*Arabidopsis thaliana*). The results demonstrated strong collinearity between wheat and barley (*Hordeum vulgare*), which contrasted sharply with reduced or absent collinearity between Triticeae and other Poaceae species, and a complete loss of synteny between Poaceae and *Arabidopsis thaliana* (Figure S5). This hierarchical pattern of conservation correlates with the phylogenetic proximity, indicating that the functional conservation of m^1^A methyltransferases gradually decreases with increasing evolutionary distance.

### 2.6. Divergent Expression Profiles of TRM6/TRM61 Homologs in Wheat

**Figure 8:**
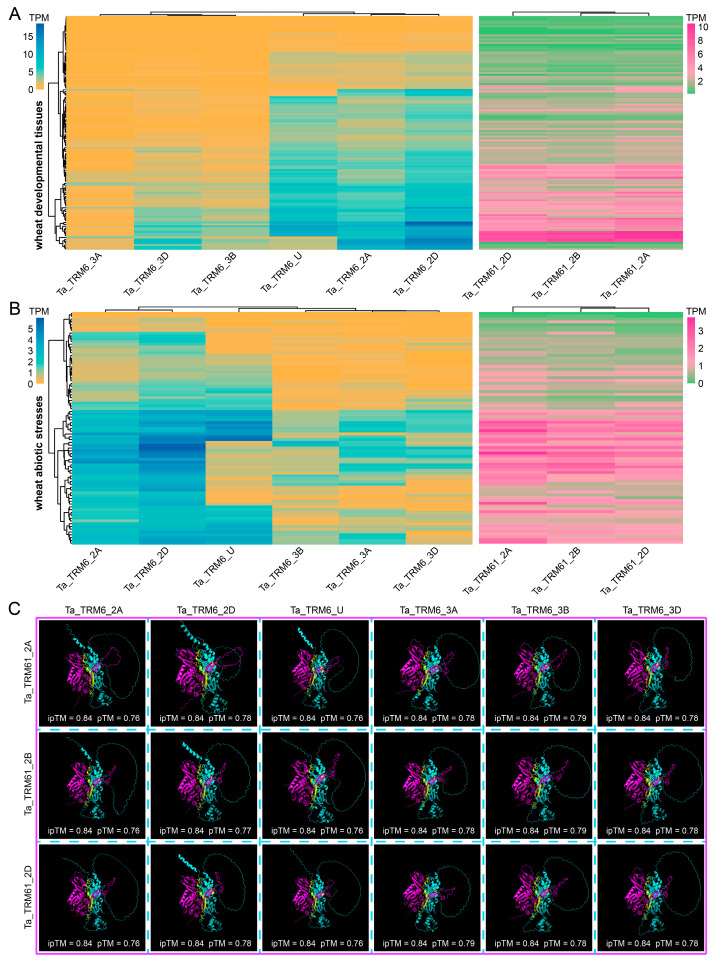
The expression profiles and protein–protein interactions between TRM6 and TRM61 homologs in wheat. (**A**,**B**) Expression patterns of *TRM6s* and *TRM61s* across different wheat (**A**) developmental tissues and (**B**) abiotic stresses. Normalized expression levels (TPM) were obtained from the WheatOmics v1.0 database (accessed on 7 March 2025). The color is correlated with the mean expression level of *TRM6* and *TRM61* genes. Source data are available in Supplementary Data S5 and S6. (**C**) Predicted interactions between Ta_TRM6 (cyan) and Ta_TRM61 (magenta) proteins. The biomolecular structure of Ta_TRM6 and Ta_TRM61 proteins and the interaction between each pair were predicted in the AlphaFold Server. pTM: the predicted template modeling score; ipTM: the interface predicted template modeling score.

The subgenome-specific gene retention in wheat results from the allohexaploidization of three diploid progenitors. To explore the expression dynamics of these accumulated *TRM6* and *TRM61* genes in hexaploid wheat, we systematically analyzed their expression profiles across various developmental tissues and under diverse abiotic stress conditions (Figure 8A,B and Supplementary Data S5 and S6). *TRM6s* exhibit expression preferences, with *Ta_TRM6_2A*, *Ta_TRM6_2D*, and *Ta_TRM6_2U* showing higher expression levels than those of the other three *TRM6* genes across both developmental stages and stress conditions. The expression profiles of the three TRM61 homologs are similar to each other under the condition of diverse abiotic stresses and exhibit similar profiles across different developmental tissues (Figure 8A,B). TRM61 and TRM6 proteins form a functional complex and function as the m^1^A methyltransferase in plants [27]. To investigate whether the differentially expressed *TRM6s* would produce proteins affecting the interactions within the TRM61/TRM6 complex through interacting with TRM61s, we used the AlphaFold Server (https://alphafoldserver.com (accessed on 4 March 2025)) to predict the binding interfaces and conformational landscapes of all possible Ta_TRM61/Ta_TRM6 heterodimers [42]. The results revealed invariant interface prediction scores (ipTM = 0.84) across all combinations (Figure 8C), indicating conserved interaction properties between TRM61 and TRM6 proteins, regardless of their differential expression patterns. However, the differential expression of these genes may affect the stoichiometry of Ta_TRM61/Ta_TRM6 complexes in vivo.

## 3. Discussion

The expanding catalog of RNA chemical modifications in plants, along with mechanistic insights into their establishment and removal, has profoundly reshaped our understanding of epitranscriptomic regulation in plant gene expression networks [3,4,5]. Identifying the “Writer-Reader-Eraser” enzyme systems that govern modification-specific regulatory circuits is crucial for deconstructing the functional hierarchy of RNA modifications. Among these, m^1^A methylation plays an indispensable regulatory role across plant growth, development, and reproductive processes, with its writer complex (TRM61/TRM6) having been preliminarily characterized in terms of function [25,27,28]. However, in contrast to extensively studied m^6^A, the biological functions and molecular mechanisms of m^1^A methylation in the plant context remain largely elusive.

In this study, we systematically identified TRM6 and TRM61 homologs across 306 plant species, encompassing evolutionary lineages from basal algae to core angiosperms. Based on this identification, we further conducted comprehensive analyses of their macroevolutionary patterns throughout the plant kingdom and interrogated their evolutionary dynamics following polyploidization in the wheat lineage. The expression level correlation between TRM6 and TRM61 homologs across phylogenetically representative land plants implies the crucial roles of m^1^A methylation in plant organogenesis and reproduction. Furthermore, our analyses suggest that *TRM6s* and *TRM61s* appear to be under selection during wheat domestication and improvement, potentially contributing to growth and developmental processes. Collectively, these findings may offer a preliminary multiscale evolutionary framework for dissecting m^1^A-mediated regulation in plants, tentatively bridging phylogenetic conservation with lineage-specific innovation in RNA modification machinery.

### 3.1. Subunit Plasticity and Structural Innovation in Plant m^1^A Methyltransferases

The m^1^A methyltransferases have been identified in several organisms. In yeast, the m^1^A modification is catalyzed by tetrameric methyltransferase composed of two types of evolutionarily related subunits (Gcd14p (TRM61) and Gcd10p (TRM6)) [34,35], whereas in the bacterium (e.g., *Thermus thermophilus*) and archaeon (e.g., *Pyrococcus abyssi*), the enzyme consists of a single subunit named TrmI (Gcd14p homolog), and functions as a homotetramer [43,44]. The human tRNA m^1^A methyltransferase exhibits both functional and structural homology with the yeast, tRNA m^1^A methyltransferase, displaying similar enzymatic activity and a conserved binary composition [45]. In *Arabidopsis*, the heterodimeric complex TRM61/TRM6 has been identified as the m^1^A methyltransferase [27]. These findings seem to imply that the subunit composition and structural features of m^1^A methyltransferases are different between eukaryotes (heterodimer) and prokaryotes (homotetramer). Evolution analysis revealed that eukaryotic *TRM6* and *TRM61* may evolve via gene duplication from prokaryotic *Trml* and undergo functional differentiation during divergent evolution [36,46]. Our phylogenomic survey of the TRM6 and TRM61 homologs across the plant kingdom revealed that these subunits of the m^1^A methyltransferase are not uniformly co-retained, with 86 phylogenetically diverse species exhibiting mutually exclusive TRM6/TRM61 ratios (0:n or n:0) spanning all major clades (Figure 1A and Supplementary Data S2). This observation implies that there is a possibility that plants may utilize prokaryotic-like homomeric TRM6 or TRM61 complexes, alongside the well-characterized TRM61/TRM6 heteromer, as functional m^1^A methyltransferases. Moreover, it remains an open question whether there are alternative m^1^A methyltransferases or functionally equivalent methylation modifications in plants, necessitating further investigation.

### 3.2. Evolutionary Conservation and Functional Divergence of TRM6 and TRM61 Homologs in Plants

In eukaryotes, TRM6 and TRM61 proteins catalyze m^1^A methylation via a conserved heterodimeric enzyme complex [27,34,35,45]. Our phylogenetic analyses reveal concordant evolutionary trajectories of TRM6 and TRM61 homologs across plant lineages (Figure 2 and Figure 3, S2 and S3), implying tight co-evolutionary constraints between these subunits. In *Arabidopsis*, *AtTRM6* and *AtTRM61* exhibit overlapping spatiotemporal expression profiles and are essential for female gametophyte and embryo development [26]. Notably, the expression levels of TRM6 and TRM61 homologs showed positive correlations (PCC > 0.5 and *p* value < 0.05) during the organogenesis and reproduction process of plants (Figure 5). These findings suggest that the evolutionarily conserved functional synergy between *TRM6* and *TRM61*, initially characterized in *Arabidopsis*, represents a shared regulatory mechanism across diverse land plant species.

Whole-genome polyploidization (WGD) followed by gene loss and diploidization is a pervasive and key driver of plant evolution [47,48,49]. The WGD event in ancestral angiosperms triggered the diversification of the regulatory genes critical for seed and flower development, thereby facilitating the rise and dominance of seed plants and angiosperms [50,51]. Genes retained after WGD exhibit nonstochastic evolutionary trajectories, with preferential retention of those encoding subunits of multimeric complexes (e.g., ribosomes, proteasomes, and transcription complexes) or components of protein interaction networks [47,52,53]. TRM6 homologs display broader copy number plasticity (0–6 copies) compared to that of TRM61 homologs (Figure 1A), suggesting more stringent purifying selection acts on the *TRM61* family. Cross-generic sequence alignment validated the conservation of TRM61 proteins, with the TRM61 protein in *Arabidopsis* showing a high degree of structural similarity to its human orthologs [26]. Structural and evolutionary analyses further elucidate that *TRM6s* and *TRM61s* in eukaryotic organisms originated via prokaryotic *TrmI* duplication, with the TRM6 protein losing catalytic residues critical for methyl donor coordination during divergence [36,46]. This loss likely reflects a functional shift that potentially enables the flexible copy number of TRM6 family members in plant genomes. Consistently, our results show that the gene structure of *TRM61s* is more conserved in wheat and its ancestral lineages than *TRM6s*, reflecting stronger purifying selection (Figure 6).

### 3.3. The Evolutionary Fate of Duplicated TRM6 and TRM61 Genes in Wheat

WGD may contribute to the variation in copy numbers of TRM6 and TRM61 homologs among the diploid progenitors of wheat [47,48,49,50,51]. Our results revealed that both *TRM6* paralogs were retained, whereas a copy of *TRM61* may have been lost during fractionation (Table 1). The stepwise allohexaploidization process, involving two sequential hybridization events, integrated ancestral TRM6 and TRM61 homologs from three diploid progenitors, ultimately establishing the 6:3 homolog ratio of modern hexaploid wheat (Table 1). The Gene Balance Hypothesis posits that stoichiometric equilibrium within multisubunit complexes determines the functional assembly integrity, thereby regulating gene expression (especially in regulatory systems) and shaping the phenotypic evolution and fitness landscapes [54]. The maintenance of dosage equilibrium provides the predominant explanatory framework for duplicated gene content restructuring [55]. The preferential expression profiles of *Ta_TRM6s* and *Ta_TRM61s* indicate that wheat flexibly modulates their gene-product stoichiometry across diverse tissues and stress conditions to maintain proper subunit ratios within the TRM6/TRM61 complex (Figure 8A,B). In *Saccharomyces cerevisiae*, reduced gene expression leads to the maintenance of functional redundancy [56], which may also be one of the reasons for the differences in the expression levels of different copies of the *TRM61* and *TRM6* genes in wheat. Overall, the evolutionary fate of duplicated *TRM6* and *TRM61* genes in wheat is shaped by the coordinated interplay among gene expression patterns, structural constraints, and functional interdependencies.

## 4. Materials and Methods

### 4.1. Species Classification and Data Collection

To identify the *TRM6* and *TRM61* gene families in plants, we retrieved proteome datasets of 306 plant species from the NCBI Genome Database (https://www.ncbi.nlm.nih.gov/genome (accessed on 20 February 2025)), Phytozome (https://jgi.doe.gov/data-and-tools/phytozome (accessed on 20 February 2025)), Ensembl Plants (http://plants.ensembl.org (accessed on 20 February 2025)) [57], 1KP (https://db.cngb.org/onekp (accessed on 20 February 2025)) [58], The *Arabidopsis* Information Resource (TAIR; https://db.cngb.org/onekp (accessed on 20 February 2025)) [59], Cucurbit Genomics Database (CuGenDB; http://cucurbitgenomics.org (accessed on 20 February 2025)) [60], Melonomics (https://www.melonomics.net (accessed on 20 February 2025)), Jatropha Genome Project (http://www.kazusa.or.jp/jatropha (accessed on 20 February 2025)), PlantGDB (https://goblinp.luddy.indiana.edu (accessed on 20 February 2025)) [61], Sol Genomics Network (SGN; https://solgenomics.net (accessed on 20 February 2025)) [62], MaizeGDB (https://maizegdb.org (accessed on 20 February 2025)) [63], Fernbase (https://fernbase.org (accessed on 20 February 2025)), GigaDB (http://gigadb.org (accessed on 20 February 2025)), PlantGenIE (https://plantgenie.org (accessed on 20 February 2025)) [64], Marchantia Polymorpha Genome Portal (MarpolBase; https://marchantia.info (accessed on 20 February 2025)) [65], and Physcomitrella patens Resource (PEATmoss; https://peatmoss.online.uni-marburg.de (accessed on 20 February 2025)) [66] (Supplementary Data S1). The downloaded data covered angiosperms (78), gymnosperms (31), ferns (25), lycophytes (14), mosses (35), hornworts (9), liverworts (23), and algae (91) (Figure S1).

### 4.2. TRM6 and TRM61 Identification

The alignment files (Stockholm format) of TRM6 (PF04189) and TRM61 (PF08704) were downloaded from the Pfam database [67]. The HMM profiles of TRM6 and TRM61 were built using the “hmmbuild” program in HMMER (v3.3) [68]. Candidate TRM6 and TRM61 homologs in plants were identified using the HMMER-based “hmmsearch” against the aforementioned proteome datasets with an E-value threshold of 1 × 10^−5^, followed by manual curation to remove redundant entries. Finally, the accuracy of the candidate proteins was checked on the HMMER web server and the NCBI Batch CD-search website [68,69]. Finalized TRM6 and TRM61 homologs were systematically renamed according to a standardized convention: phylum_species abbreviation_family-serial (e.g., Angiosperm*_A*.*thaliana_TRM6-1*). The species abbreviation combines the capitalized genus initial with the full species name (e.g., *Arabidopsis thaliana* abbreviated to *A*.*thaliana*), while the serial number reflects species-specific gene counts (Supplementary Data S2).

### 4.3. Phylogenetic Analysis of the TRM6 and TRM61 Families

The multiple protein sequence alignments were generated using PRANK software [70]. The alignment results were subsequently trimmed with Trimal (v1.2) (−gt 0.1) and subjected to evolutionary model selection using ModelFinder [71,72]. Maximum likelihood (ML) phylogenetic trees were constructed with IQ-TREE (v2.1.3), incorporating 1500 replicates of ultrafast bootstrap approximation and SH-aLRT tests for branch support evaluation [73,74]. The branch lengths were extracted from the tree file using the ape (v5.0) R package [75]. The final tree visualization was conducted through the iTOL v7 platform [76].

### 4.4. Expression Pattern and Conserved Motif Analysis of TRM6 and TRM61 Homologs in Phylogenetically Representative Plants

To investigate the contribution of the *TRM6* and *TRM61* gene families to plant organogenesis and reproduction, we retrieved the expression data across 13 phylogenetically representative plant species under diverse tissue and stress conditions from EVOREPRO (www.evorepro.plant.tools (accessed on 27 February 2025)) and MaizeGDB (accessed on 27 February 2025) [63,77] (Supplementary Data S3). The selected species spanned key evolutionary lineages: algae (*Cyanophora paradoxa* and *Chlamydomonas reinhardtii*), bryophytes (*Marchantia polymorpha* and *Physcomitrium patens*), lycophytes (*Selaginella moellendorffii*), gymnosperms (*Ginkgo biloba* and *Picea abies*), basal angiosperms (*Amborella trichopoda*), monocots (*Zea mays* and *Oryza sativa*), and eudicots (*Arabidopsis thaliana*, *Solanum lycopersicum* and *Vitis vinifera*). We then systematically analyzed the expression patterns to elucidate their roles in organ development and reproductive evolution during land plant diversification. The sequences of TRM6 and TRM61 proteins in these 13 plants were further submitted to the MEME Suite (https://meme-suite.org/ (accessed on 18 March 2025)) for motif discovery [78].

### 4.5. The Evolutionary Dynamics Analysis of TRM6 and TRM61 Families in the Wheat System

The identified TRM6 and TRM61 homologs in wheat and its diploid and tetraploid progenitors were extracted for evolutionary dynamics analysis. To clarify the chromosomal evolutionary relationships of *TRM6s* and *TRM61s* in hexaploid wheat, we implemented a hierarchical nomenclature system (Species Prefix_TRM family_Chromosome) for all identified genes across wheat and its diploid (*T. urartu* and *A.tauschii*) and tetraploid (*T.dicoccoides*) ancestral lineages. The species-specific prefixes were designated as *Ta* for hexaploid wheat (*T. aestivum*), *Td* for tetraploid wheat (*T. dicoccoides*), *Tu* for diploid *T. urartu*, and *Aet* for diploid *A. tauschii*, followed by the TRM family name and subgenome-specific chromosomal localization (e.g., *Ta_TRM6_2D* for the *TRM6* gene on wheat chromosome 2D). The phylogenetic analysis of TRM6 and TRM61 proteins in wheat and its diploid and tetraploid progenitors was conducted as described above. The grand average of hydropathicity (GRAVY), theoretical isoelectric point (pI), and molecular weight (Mw) were predicted in the online ProtParam tool (https://web.expasy.org/protparam/ (accessed on 20 March 2025)) [79].

The exon–intron structures of *TRM6s* and *TRM61s* were extracted from the annotation files of the four species and were displayed using the Gene Structure Display Server (GSDS v2.0) [80,81,82,83,84]. The *cis*-acting regulatory elements in the promoter region (2000 bp of each gene) were analyzed in the PlantCARE database (accessed on 3 March 2025) [85]. The SNP information of the 5′ and 3′ UTR variant, missense variant, splice region variant, and their allele frequency distribution pattern in five ploidy wheat groups of these *Ta_TRM6s* and *Ta_TRM61s* were obtained in the Wheat Genome Variation Database (WGVD) dataset for selective footprint analysis (accessed on 11 March 2025) [86] (Supplementary Data S4). The microcollinearity profiles of *TRM6s* and *TRM61s* across wheat and *Arabidopsis thaliana* (TAIR10), *Zea mays* (B73 RefGen v4), *Sorghum bicolor* (NCBIv3), *Oryza sativa Japonica* (IRGSP 1.0), and *Hordeum vulgare* (Pangenome ZDM01467) were conducted in Triticeae-GeneTribe (http://wheat.cau.edu.cn/TGT/ (accessed on 11 March 2025)) [87].

### 4.6. The Expression Profile Analysis and Interaction Prediction of TRM6 and TRM61 Homologs in Wheat

The normalized expression data (TPM) of *Ta_TRM6s* and *Ta_TRM61s* across different development tissues and under abiotic stresses were obtained from the GeneExpression tool in WheatOmics v1.0 (accessed on 7 March 2025) [88] (Supplementary Data S5 and S6). The protein–protein interactions between each Ta_TRM6 and Ta_TRM61 protein in wheat were predicted using AlphaFold3 via the AlphaFold Server website (https://alphafoldserver.com (accessed on 4 March 2025)) [42]. The resulting three-dimensional structures of protein interactions were visualized using PyMOL (v3.1) software [89].

### 4.7. Statistical Analysis

The expression level correlation between *TRM6s* and *TRM61s* in each phylogenetically representative plant species was assessed using PCC. The statistical significance of Pearson correlation was assessed using two-tailed *p* values with 95% confidence intervals, without multiplicity correction for pairwise comparisons.

## 5. Conclusions

This study systematically characterizes the m^1^A methyltransferase subunits, TRM6 and TRM61, across plant evolution, delineating their macroevolutionary conservation from algae to angiosperms and their adaptive diversification in wheat. The *TRM6* and *TRM61* families exhibit lineage-specific expansions associated with major transitions in plant diversification, while maintaining conserved motifs and displaying comparable expression profiles in the organogenesis and reproduction processes of plants. In wheat and its progenitors, the TRM6 and TRM61 proteins demonstrate coordinated evolutionary patterns that align with polyploidization events during allopolyploid speciation. The allelic frequency dynamics of the *TRM6* and *TRM61* genes during wheat domestication and improvement appear to correlate with the potential involvement of m^1^A methylation in mediating environmental adaptation processes and yield-related trait optimization. Furthermore, both gene families exhibit diverse expression profiles across developmental tissues and under various abiotic stress conditions. Overall, this work deciphers the evolution of plant m^1^A writers, establishing a framework to investigate their developmental and stress-responsive roles, aiding crop improvement strategies.

## Figures and Tables

**Table 1:** The TRM6 and TRM61 homologs in wheat and its diploid and tetraploid progenitors. The calculation of hydropathicity (GRAVY), theoretical isoelectric point (pI), and molecular weight (Mw) is presented in the Section 4.

Species	Transcript ID	Gene Name	Protein Length	Mw	pI	GRAVY
*T. urartu*	TuG1812S0000898200.01.P01	*Tu_TRM6_S1* ^1^	351	37,991.54	7.66	−0.144
*T. urartu*	TuG1812S0002603300.01.P01	*Tu_TRM6_S2*	511	54,931.29	5.43	−0.273
*A. tauschii*	AET2Gv20006900.9	*Aet_TRM6_2D*	480	51,766.75	5.5	−0.243
*A. tauschii*	AET3Gv20046100.2	*Aet_TRM6_3D*	475	50,921.88	6.12	−0.235
*T. dicoccoides*	TRIDC2AG000170.1	*Td_TRM6_2A*	294	31,273.83	4.56	−0.458
*T. dicoccoides*	TRIDC3AG000690.1	*Td_TRM6_3A*	356	37,786.76	5.18	−0.242
*T. dicoccoides*	TRIDC3BG003460.1	*Td_TRM6_3B*	476	51,160.16	8.89	−0.364
*T. aestivum*	TraesCS2A02G004800.1	*Ta_TRM6_2A*	507	54,463.62	5.29	−0.286
*T. aestivum*	TraesCS2D02G004000.1	*Ta_TRM6_2D*	483	51,921.91	5.61	−0.263
*T. aestivum*	TraesCS3A02G024400.1	*Ta_TRM6_3A*	473	50,831.81	5.96	−0.208
*T. aestivum*	TraesCS3B02G020400.1	*Ta_TRM6_3B*	474	50,670.67	5.81	−0.194
*T. aestivum*	TraesCS3D02G021800.1	*Ta_TRM6_3D*	481	51,585.71	5.96	−0.197
*T. aestivum*	TraesCSU02G012500.1	*Ta_TRM6_U* ^2^	517	55,236.48	5.42	−0.269
*T. urartu*	TuG1812G0200003197.01.P01	*Tu_TRM61_2A*	335	35,973.76	7.61	−0.106
*A. tauschii*	AET2Gv20616500.1	*Aet_TRM61_2D*	336	35,939.64	7.13	−0.124
*T. dicoccoides*	TRIDC2AG039430.1	*Td_TRM61_2A*	336	35,982.77	7.62	−0.105
*T. dicoccoides*	TRIDC2BG042450.1	*Td_TRM61_2B*	336	35,925.65	7.64	−0.113
*T. aestivum*	TraesCS2A02G273600.1	*Ta_TRM61_2A*	335	35,982.77	7.62	−0.105
*T. aestivum*	TraesCS2B02G291400.1	*Ta_TRM61_2B*	335	35,939.68	7.64	−0.114
*T. aestivum*	TraesCS2D02G272600.1	*Ta_TRM61_2D*	335	35,939.64	7.13	−0.124

^1^ In the gene identifiers, *Tu_TRM6_S1* and *Tu_TRM6_S1*, the suffix “S” denotes the scaffold designation in the reference genome assembly. ^2^ In the gene identifier, *Ta_TRM6_U*, the suffix “U” indicates that chromosomal assignment information is currently unknown.

## Data Availability

The original contributions presented in this study are included in the article/Supplementary Material. Further inquiries can be directed to the corresponding author.

## Supplementary Materials

The following supporting information can be downloaded at: https://www.mdpi.com/article/10.3390/plants14121778/s1, Figure S1: The number of species across different taxonomies used for the identification of TRM6 and TRM61 homologs in plants; Figure S2: Phylogenetic tree of TRM6 homologs in plants, with branch lengths displayed; Figure S3: Phylogenetic tree of TRM61 homologs in plants, with branch lengths displayed; Figure S4: The expression patterns of *TRM6* and *TRM61* genes in (A) *Chlamydomonas reinhardtii*, (B) *Cyanophora paradoxa*, (C) *Solanum lycopersicum*, (D) *Vitis vinifera*, and (E) *Picea abies*; Figure S5: The microcollinearity patterns of *TRM6s* and *TRM61s*. Supplementary Data S1: List of plant species employed for the identification and evolutionary analysis of TRM6 and TRM61 homologs; Supplementary Data S2: TRM6 and TRM61 homologs identified from 306 plant species; Supplementary Data S3: The expression levels of *TRM6* and *TRM61* genes in 13 phylogenetically representative species; Supplementary Data S4. Selective footprints of *Ta_TRM6s* and *Ta_TRM61s* during the domestication and improvement process of wheat. Supplementary Data S5: The expression levels of *Ta_TRM6* and *Ta_TRM61* genes in wheat abiotic stress responses; Supplementary Data S6: The expression levels of *Ta_TRM6* and *Ta_TRM61* genes across diverse developmental tissues of wheat.
