# Supplementary material for: Evolutionary Dynamics of Plant TRM6/TRM61 Complexes
Source: Plants (Basel). 2025 Jun 11;14(12):1778. doi: 10.3390/plants14121778 (PMC12196605; doi:10.3390/plants14121778)
Supplement: Supplementary file 1 [file plants-14-01778-s001.zip › Supplementary Figures.pdf]

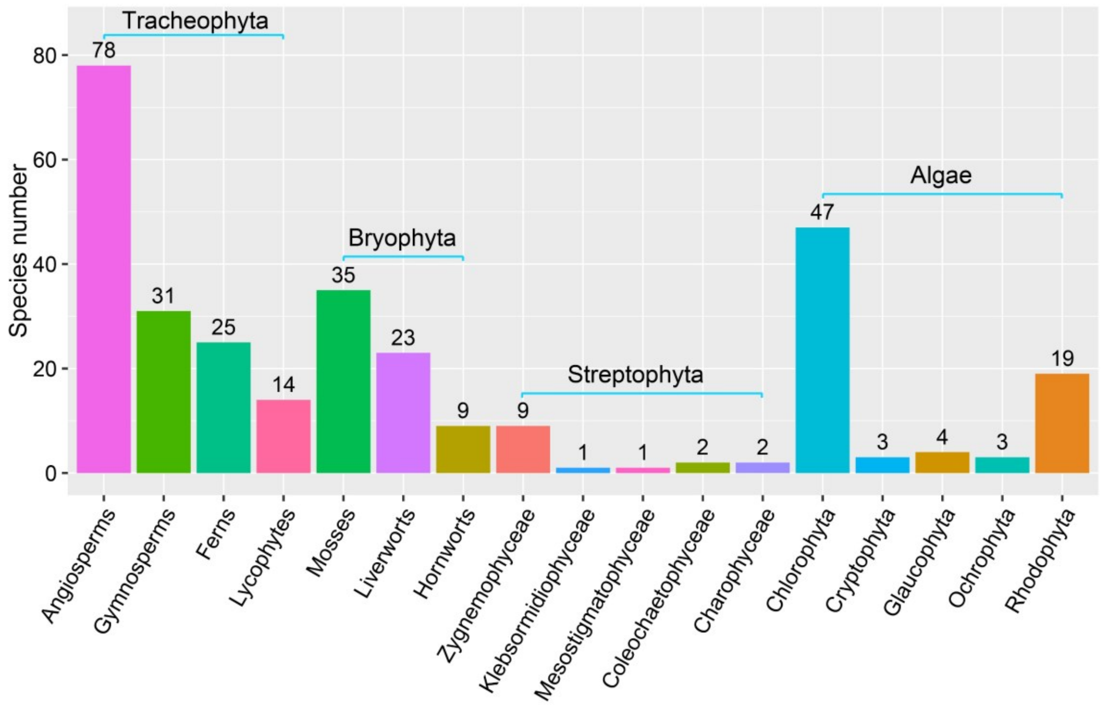

Figure S1. The number of species across different taxonomies used for the identification of TRM6 and TRM61 homologs in plants. The list of these species is provided in Supplementary Data 1.

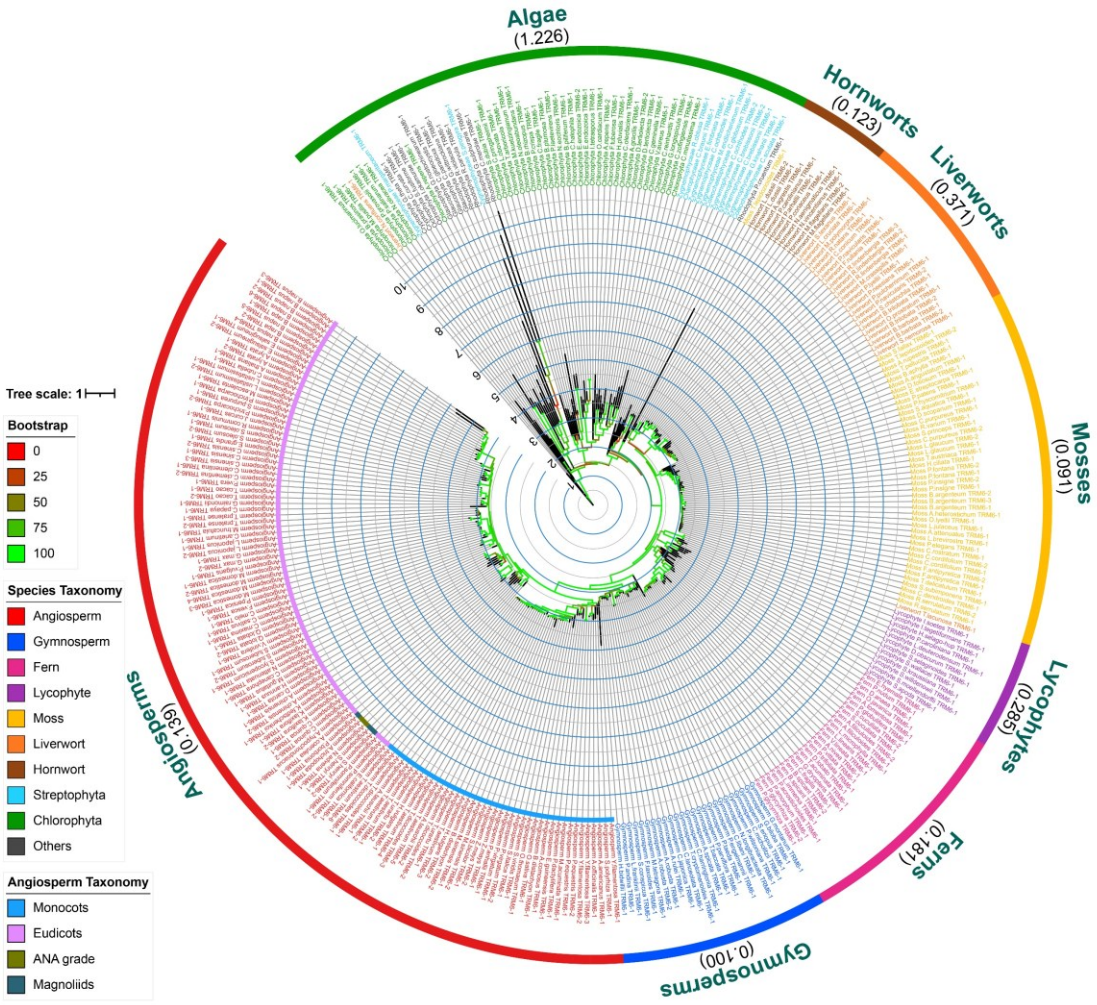

Figure S2. Phylogenetic tree of TRM6 homologs in plants, with branch lengths displayed. Tree construction followed the same methods as outlined in Figure 2.

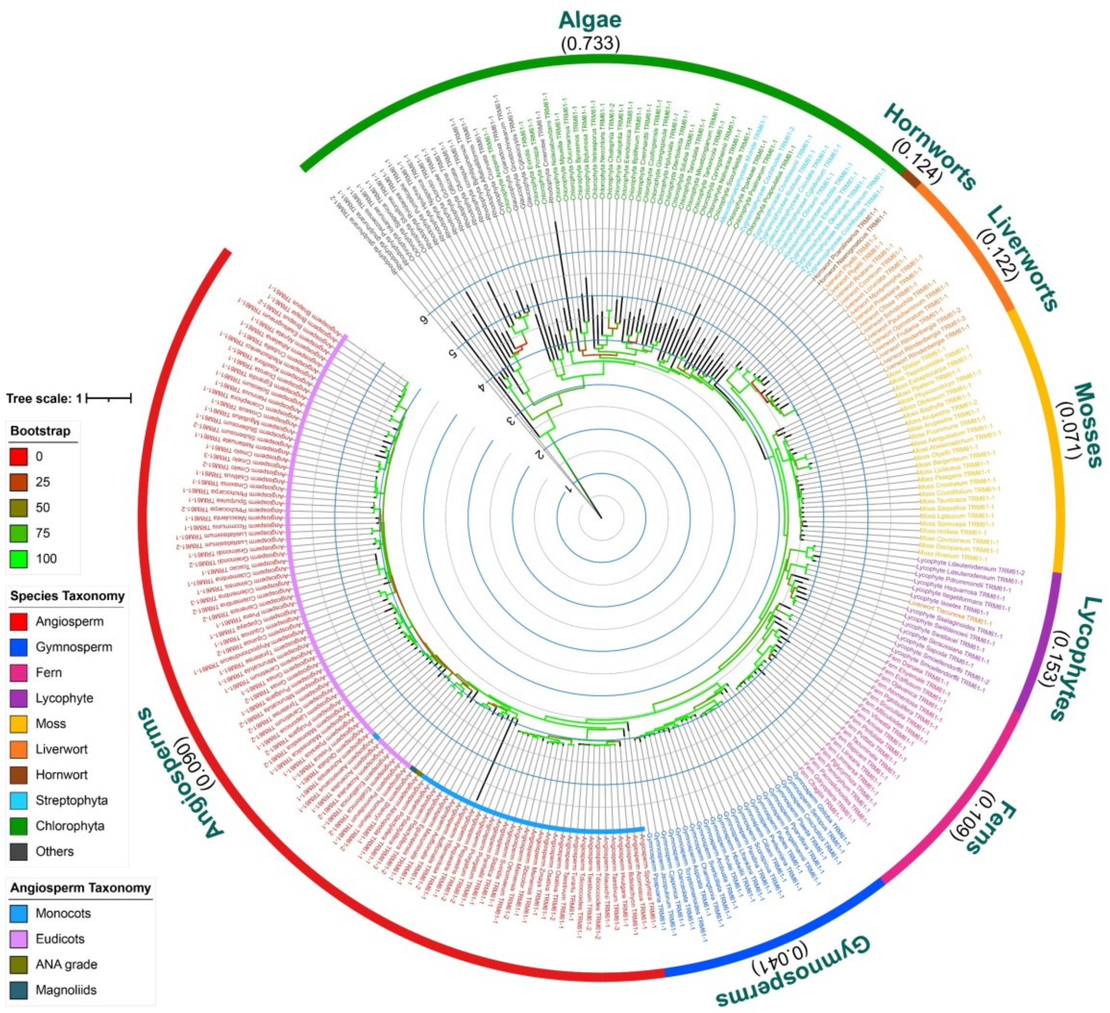

Figure S3. Phylogenetic tree of TRM61 homologs in plants, with branch lengths displayed. Tree construction followed the same methods as outlined in Figure 2.

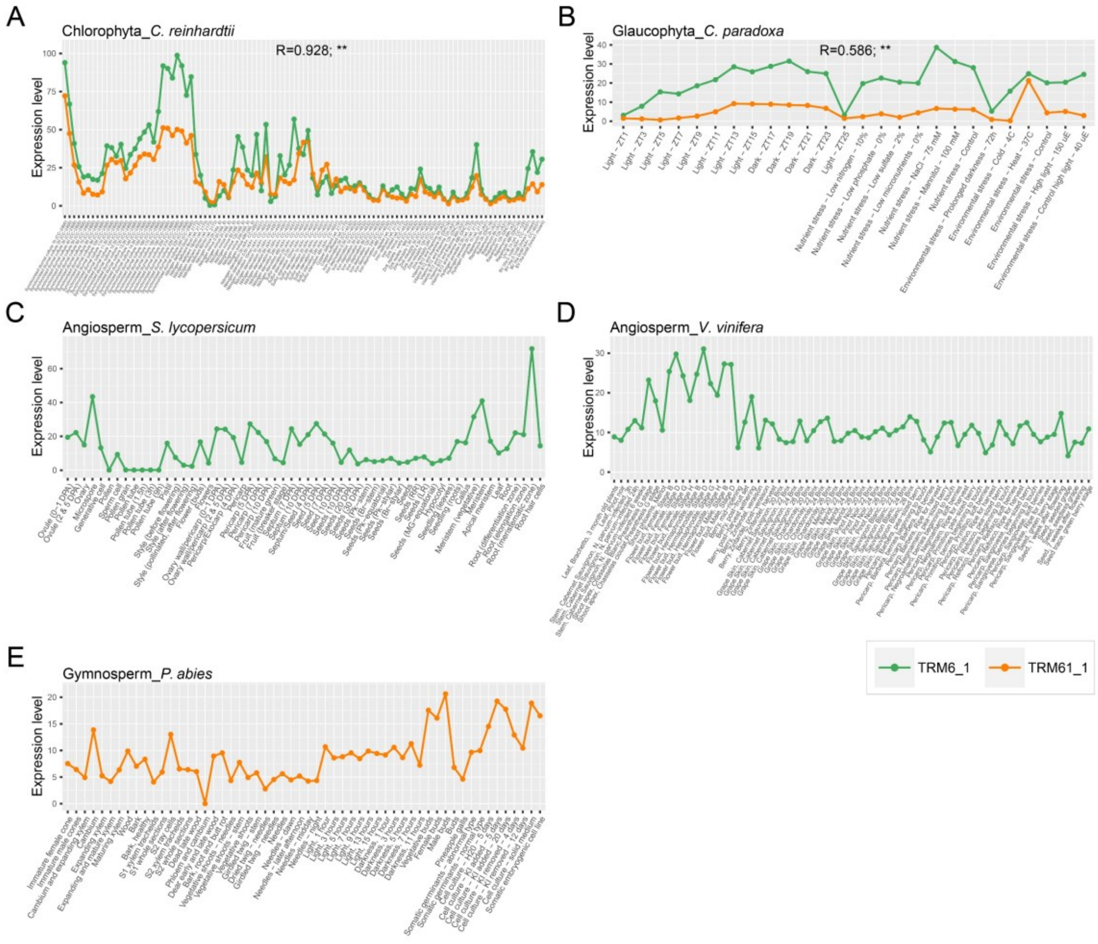

Figure S4. The expression patterns of *TRM6* and *TRM61* genes in **(A)** *Chlamydomonas reinhardtii*, **(B)** *Cyanophora paradoxa*, **(C)** *Solanum lycopersicum*, **(D)** *Vitis vinifera*, and **(E)** *Picea abies*. Both the data acquisition procedures and the significance test of the expression level differences between *TRM6* and *TRM61* genes follow the same methods as detailed in Figure 5.

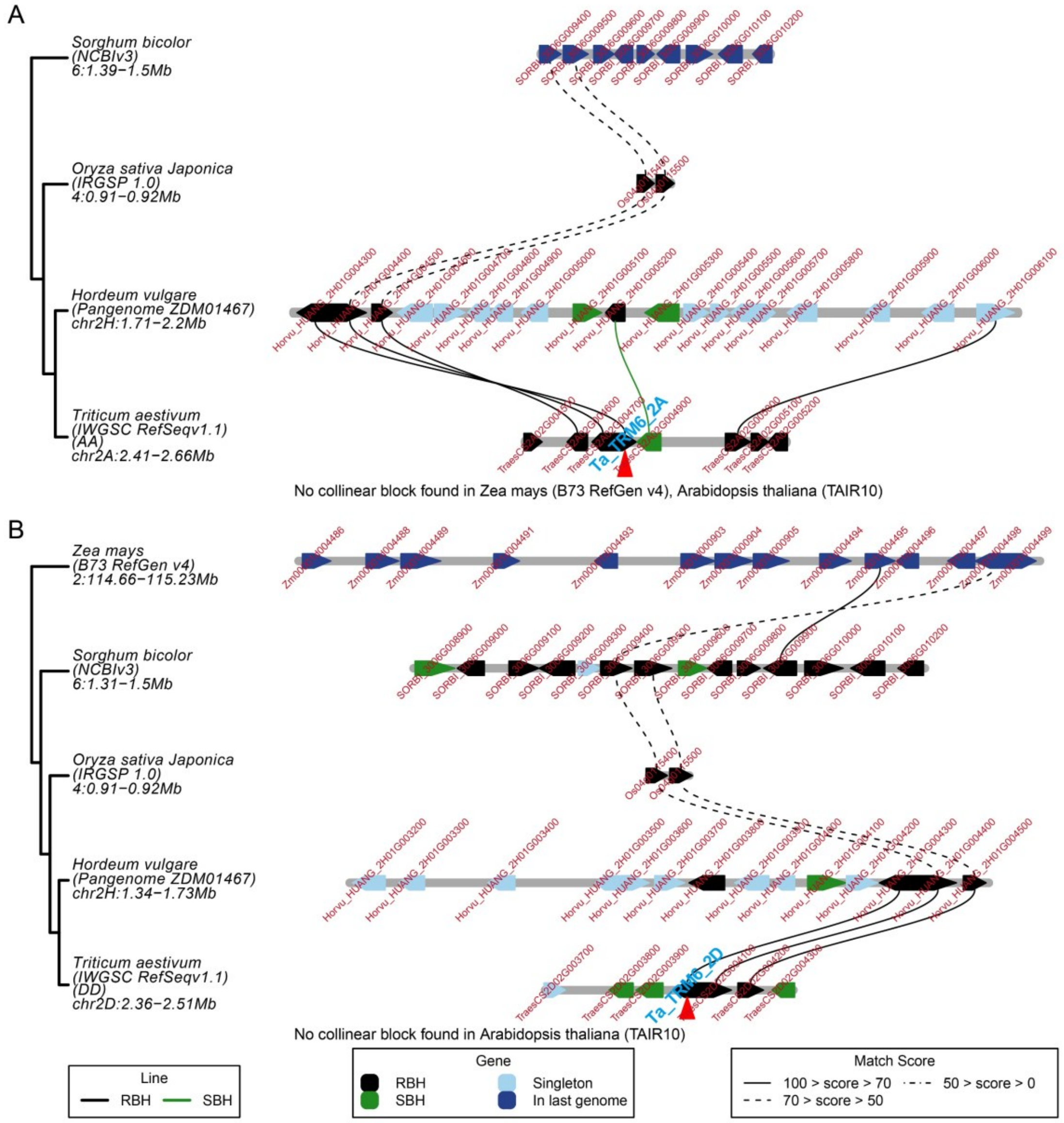

Figure S5. The microcollinearity patterns of *TRM6s* and *TRM61s*. **(A-H)** The microcollinearity patterns of TRM6s and TRM61s across wheat and *Arabidopsis thaliana* (TAIR10), *Zea mays* (B73 RefGen v4), *Sorghum bicolor* (NCBIv3), *Oryza sativa Japonica* (IRGSP 1.0) and *Hordeum vulgare* (Pangenome ZDM01467). The collinearity profiles were generated using Triticeae-GeneTribe (http://wheat.cau.edu.cn/TGT/).

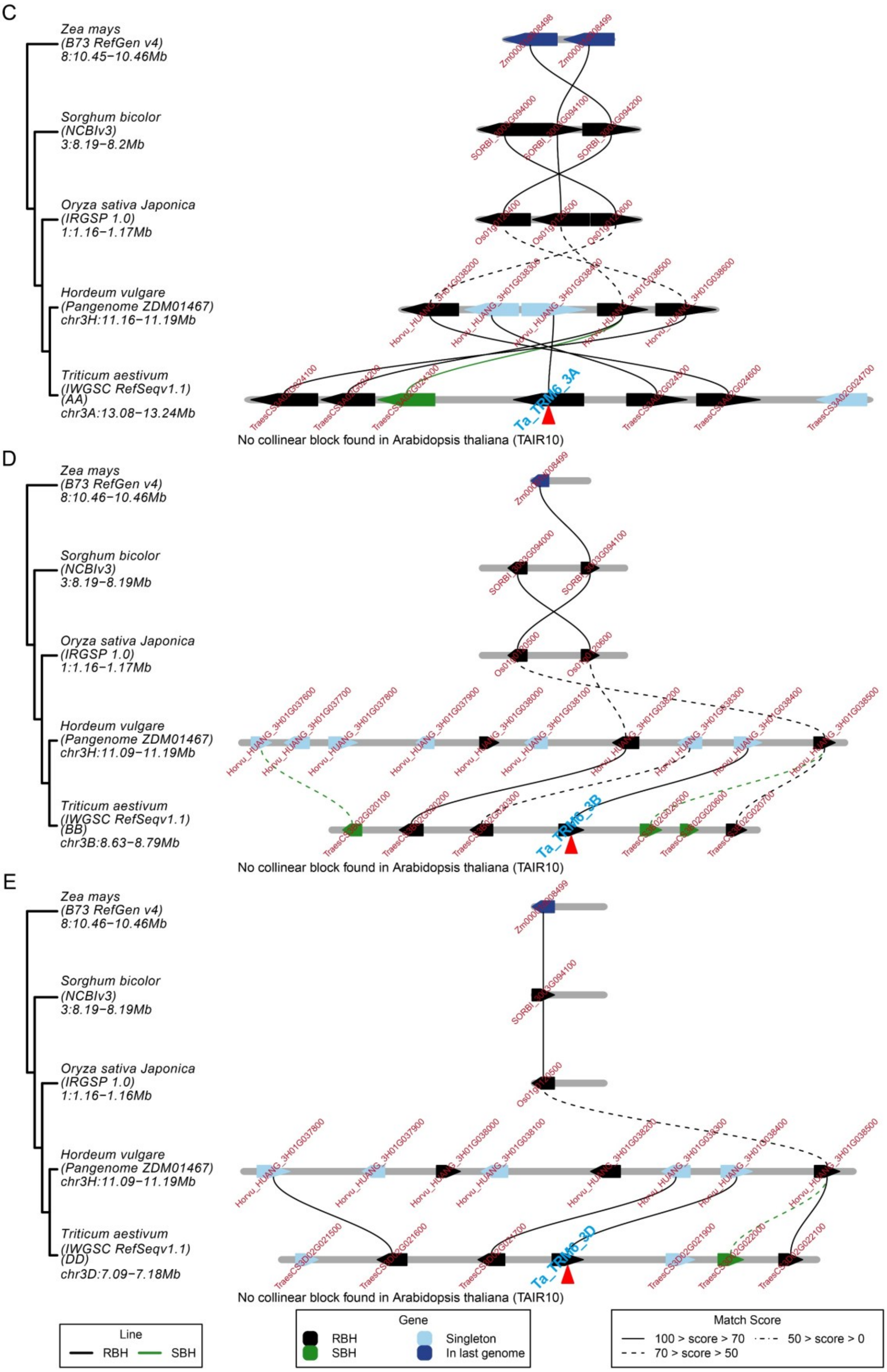
C
Zea mays
(B73 RefGen v4)
8:10.45−10.46Mb
Sorghum bicolor
(NCBIv3)
3:8.19−8.2Mb
Oryza sativa Japonica
(IRGSP 1.0)
1:1.16−1.17Mb
Hordeum vulgare
(Pangenome ZDM01467)
chr3H:11.16−11.19Mb
Triticum aestivum
(IWGSC RefSeqv1.1)
(AA)
chr3A:13.08−13.24Mb
Ta_TRM6_3A
No collinear block found in Arabidopsis thaliana (TAIR10)
D
Zea mays
(B73 RefGen v4)
8:10.46−10.46Mb
Sorghum bicolor
(NCBIv3)
3:8.19−8.19Mb
Oryza sativa Japonica
(IRGSP 1.0)
1:1.16−1.17Mb
Hordeum vulgare
(Pangenome ZDM01467)
chr3H:11.09−11.19Mb
Triticum aestivum
(IWGSC RefSeqv1.1)
(BB)
chr3B:8.63−8.79Mb
Ta_TRM6_3B
No collinear block found in Arabidopsis thaliana (TAIR10)
E
Zea mays
(B73 RefGen v4)
8:10.46−10.46Mb
Sorghum bicolor
(NCBIv3)
3:8.19−8.19Mb
Oryza sativa Japonica
(IRGSP 1.0)
1:1.16−1.16Mb
Hordeum vulgare
(Pangenome ZDM01467)
chr3H:11.09−11.19Mb
Triticum aestivum
(IWGSC RefSeqv1.1)
(DD)
chr3D:7.09−7.18Mb
Ta_TRM6_3D
No collinear block found in Arabidopsis thaliana (TAIR10)
Line
RBH
SBH
Gene
RBH
SBH
Singleton
In last genome
Match Score
100 > score > 70
70 > score > 50
50 > score > 0

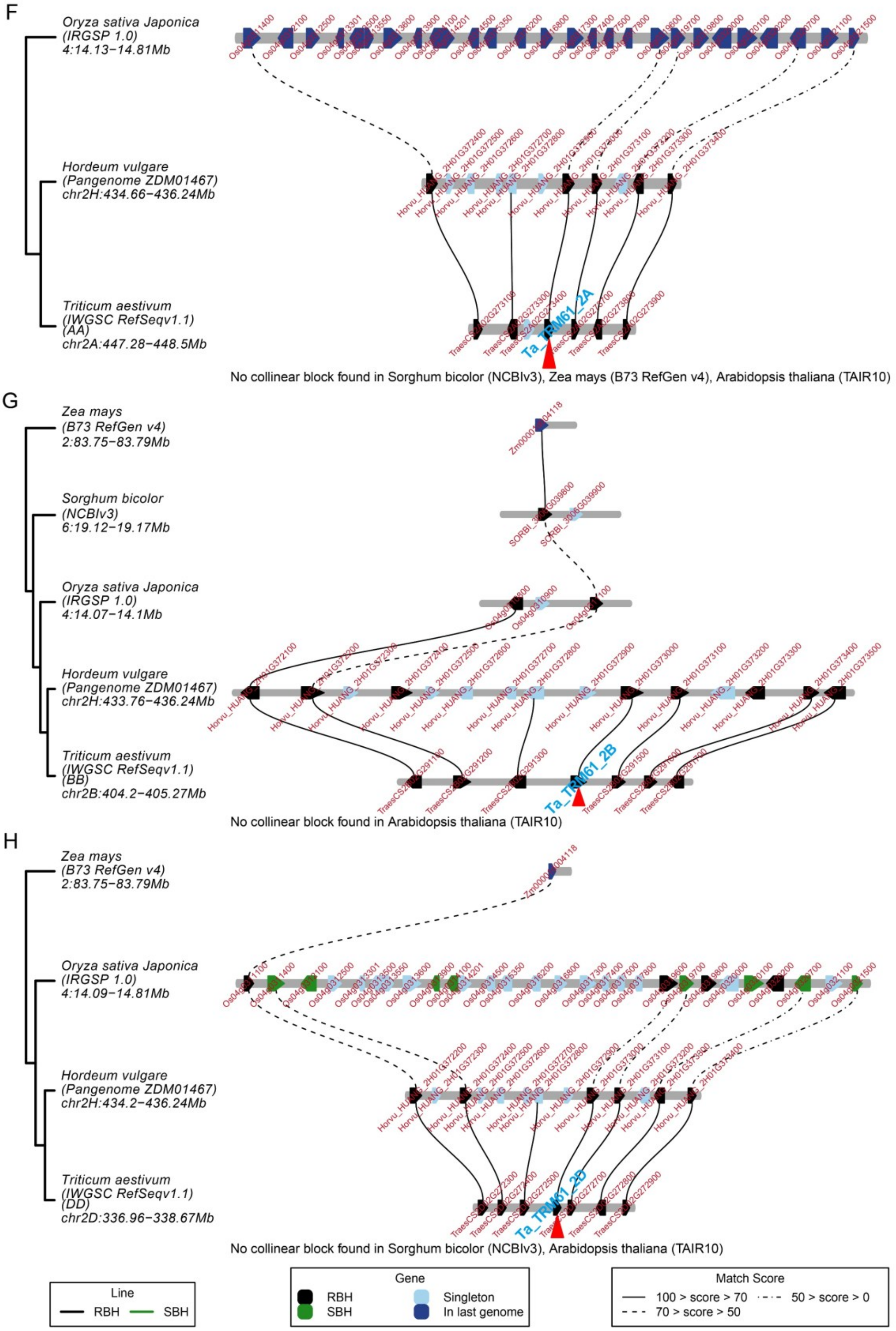

F
Oryza sativa Japonica (IRGSP 1.0) 4:14.13−14.81Mb
Hordeum vulgare (Pangenome ZDM01467) chr2H:434.66−436.24Mb
Triticum aestivum (IWGSC RefSeqv1.1) (AA) chr2A:447.28−448.5Mb
Ta_TRM61_2A
No collinear block found in Sorghum bicolor (NCBIv3), Zea mays (B73 RefGen v4), Arabidopsis thaliana (TAIR10)
G
Zea mays (B73 RefGen v4) 2:83.75−83.79Mb
Sorghum bicolor (NCBIv3) 6:19.12−19.17Mb
Oryza sativa Japonica (IRGSP 1.0) 4:14.07−14.1Mb
Hordeum vulgare (Pangenome ZDM01467) chr2H:433.76−436.24Mb
Triticum aestivum (IWGSC RefSeqv1.1) (BB) chr2B:404.2−405.27Mb
Ta_TRM61_2B
No collinear block found in Arabidopsis thaliana (TAIR10)
H
Zea mays (B73 RefGen v4) 2:83.75−83.79Mb
Oryza sativa Japonica (IRGSP 1.0) 4:14.09−14.81Mb
Hordeum vulgare (Pangenome ZDM01467) chr2H:434.2−436.24Mb
Triticum aestivum (IWGSC RefSeqv1.1) (DD) chr2D:336.96−338.67Mb
Ta_TRM61_2D
No collinear block found in Sorghum bicolor (NCBIv3), Arabidopsis thaliana (TAIR10)
Line
RBH
SBH
Gene
RBH
SBH
Singleton
In last genome
Match Score
100 > score > 70
70 > score > 50
50 > score > 0

Supplementary Data

Supplementary Data S1. List of plant species employed for the identification and evolutionary analysis of TRM6 and TRM61 homologs.

Supplementary Data S2. TRM6 and TRM61 homologs identified from 306 plant species. Each of the identified genes was renamed according to the rules as described in the Materials and Methods section. The species that possess only one type of TRM6 or TRM61 homolog (with the ratio of TRM6:TRM61 = 0:n or n:0) or lack both gene homologs are listed in Sheet2.

Supplementary Data S3. The expression levels of *TRM6* and *TRM61* genes in 13 phylogenetically representative species. These species include *Arabidopsis thaliana*, *Amborella trichopoda*, *Ginkgo biloba*, *Zea mays*, *Marchantia polymorpha*, *Physcomitrium patens*, *Picea abies*, *Oryza sativa*, *Selaginella moellendorffii*, *Solanum lycopersicum*, *Vitis vinifera*, *Chlamydomonas reinhardtii* and *Cyanophora paradoxa*. The expression levels of *TRM6* and *TRM61* genes were obtained from the EVOREPRO (www.evorepro.plant.tools) and MaizeGDB (https://maizegdb.org/) databases. The gene expression levels of each of these 13 species are listed in separate sheets.

Supplementary Data S4. Selective footprints of *Ta_TRM6s* and *Ta_TRM61s* during the domestication and improvement process of wheat. SNP variants (5'/3' UTR, missense, splice regions) and their allele frequencies across five wheat lineage groups were retrieved from the Wheat Genome Variation Database (WGVD).

Supplementary Data S5. The expression levels of *Ta_TRM6* and *Ta_TRM61* genes in wheat abiotic stress responses. The normalized expression levels (TPM) of each gene were retrieved from the WheatOmics database (http://wheatomics.sdau.edu.cn/).

Supplementary Data S6. The expression levels of *Ta_TRM6* and *Ta_TRM61* genes across diverse developmental tissues of wheat. The normalized expression levels (TPM) for each gene were retrieved from the WheatOmics database (http://wheatomics.sdau.edu.cn/).
